# Unmasking Wilson Disease: A Rare Paediatric Case of Haemolysis and Hepatic Dysfunction Without Neurological Features

**DOI:** 10.7759/cureus.77726

**Published:** 2025-01-20

**Authors:** Rim Al Zohbi, Issa Awaida, Solay Farhat, Fatima Ghandour

**Affiliations:** 1 Department of Paediatrics, Lebanese University, Faculty of Medical Sciences, Beirut, LBN; 2 Department of Paediatrics, Al Zahraa Hospital University Medical Center, Beirut, LBN

**Keywords:** copper accumulation, hepatosplenomegaly in infancy, liver cirrhosis, pediatrics, wilson`s disease

## Abstract

Wilson disease (WD) is a rare autosomal recessive disorder characterized by abnormal copper accumulation in various organs, including the liver, brain, and kidneys. Its diverse clinical presentation, particularly in paediatric patients, poses a significant diagnostic challenge. We present a unique case of a 10-year-old boy initially misdiagnosed with immune thrombocytopenic purpura (ITP) and haemolytic anaemia, later diagnosed with WD after extensive evaluation. The patient’s condition progressed to liver cirrhosis, spontaneous bacterial peritonitis, acute respiratory distress syndrome, and multisystem organ failure despite prompt initiation of copper-chelating therapy. This case underscores the importance of considering WD in paediatric patients with unexplained liver dysfunction, haemolysis, or hepatosplenomegaly, even in the absence of classic neurological symptoms or Kayser-Fleischer rings. The Wilson disease scoring system proved valuable in this atypical presentation, guiding diagnosis and management. This report highlights the need for heightened awareness, early diagnosis, and a multidisciplinary approach in managing WD, particularly in its advanced stages.

## Introduction

Wilson disease (WD), also known as hepatolenticular degeneration, is a rare autosomal recessive genetic disorder characterized by the pathological accumulation of copper in multiple organs and tissues, including the liver, brain, kidneys, and corneas [1]. This condition results from mutations in the *ATP7B* gene, which encodes a copper-transporting ATPase enzyme responsible for copper export from the liver into bile for excretion and its incorporation into ceruloplasmin, a copper-binding protein in the bloodstream [1,2]. Mutations in *ATP7B* impair these functions, leading to copper accumulation in the liver, progressive hepatocellular damage, and subsequent copper deposition in extrahepatic tissues [2]. With an estimated global prevalence of 1 in 10,000 to 30,000 individuals [3], the prevalence rates of WD can be significantly higher in regions with high consanguinity, such as isolated populations in Crete being as common as 1 in 15 live births [4]. In fact, the carrier frequency for mutations in the *ATP7B* gene, which underlies the disease, is approximately 1 in 90 individuals [3], with fulminant presentations being more frequently observed in females [3,5].

The clinical spectrum of Wilson disease is highly heterogeneous and can present at any age, typically between 5 and 35 years, with a mean age of onset around 13 years [6]. Common hepatic manifestations include asymptomatic liver enzyme abnormalities, hepatomegaly, jaundice, cirrhosis, or fulminant liver failure [7]. Neurological symptoms, more prevalent in older children and adults, include tremors, dystonia, dysarthria, and psychiatric conditions such as depression, anxiety, or psychosis [8]. A hallmark finding, particularly in cases with neurological involvement, is the presence of Kayser-Fleischer rings, brownish corneal copper deposits visible upon slit-lamp examination [8]. Despite these hallmark signs, atypical presentations such as isolated haematological abnormalities, including haemolytic anaemia and thrombocytopenia, are frequently overlooked and can lead to misdiagnosis.

Indeed, diagnosing Wilson disease poses significant challenges due to its diverse clinical manifestations and nonspecific early symptoms, which can mimic conditions such as autoimmune hepatitis, primary sclerosing cholangitis, haemolytic anaemia, and immune thrombocytopenic purpura (ITP) [9]. A comprehensive diagnostic approach incorporating clinical assessment, biochemical tests, specifically serum ceruloplasmin levels, 24-hour urinary copper excretion, and hepatic copper quantification, with genetic testing for *ATP7B* mutations, is essential for accurate diagnosis [10]. Indeed, the Wilson disease scoring system, which integrates these modalities, provides an additional structured framework to guide diagnosis and is particularly useful in atypical cases or paediatric populations where classic features, such as Kayser-Fleischer rings, are often absent [11,12]. This system evaluates a combination of biochemical, clinical, and genetic findings, offering a systematic and evidence-based approach to diagnosis, even in challenging presentations.

Paediatric cases of Wilson disease often present with hepatic symptoms, such as hepatomegaly and jaundice, but lack classic neurological features. Moreover, isolated haematological findings, including haemolytic anaemia or thrombocytopenia, as seen in this case, can obscure diagnosis and delay treatment. The overlap of these symptoms with other conditions highlights the importance of maintaining a high index of suspicion for WD in children presenting with unexplained hepatic, haematological, or neuropsychiatric abnormalities. Our case report highlights an unusual presentation of Wilson disease in a 10-year-old boy initially misdiagnosed with ITP and haemolytic anaemia. This case underscores the need to maintain a high index of suspicion for Wilson disease in paediatric patients presenting with overlapping hepatic, haematologic, and neuropsychiatric abnormalities, emphasizing the importance of the Wilson disease scoring system in guiding diagnostic decisions, with the need for early identification and intervention to prevent irreversible complications.

## Case presentation

We are presenting a case of a 10-year-old boy who presented to our hospital with a four-month history of petechiae, progressive abdominal distension, fatigue, and laboratory findings indicative of haemolytic anemia. The child had been in good health until four months prior when his parents observed the onset of petechiae, primarily localized to his lower extremities, accompanied by fatigue and abdominal distension. There was no history of prior hospitalizations, surgeries, or consanguinity in the family. However, a paternal cousin had died of liver disease during childhood. Physical examination revealed hepatosplenomegaly and petechiae, prompting referral to a haematologist for further evaluation.

Initial laboratory investigations revealed significant abnormalities (Table 1).

**Table 1:** Initial laboratory findings on presentation.

Parameter	Value	Reference range	Interpretation
Aspartate aminotransferase (AST)	3000 IU/L	10-40 IU/L	Markedly elevated, indicating liver injury
Alanine aminotransferase (ALT)	1500 IU/L	7-56 IU/L	Markedly elevated, indicating liver injury
Lactate dehydrogenase (LDH)	1300 IU/L	140-280 IU/L	Elevated, consistent with haemolysis
Haptoglobin	<8 mg/dL	30-200 mg/dL	Decreased, consistent with haemolysis
Platelets	79 × 10³/µL	150-400×10³/µL	Thrombocytopenia
Haemoglobin	11.9	12.0-16.0 g/dL (female)/13.5-17.5 g/dL (male)	Mildly decreased, indicating possible anaemia
Prothrombin time (PT)	19.4 sec	11-15 sec	Prolonged, indicating coagulopathy
International normalized ratio (INR)	1.84	0.8-1.2	Prolonged, indicating coagulopathy
Creatinine	0.39 mg/dL	0.6-1.2 mg/dL	Compromised with respect to age

Liver function tests (LFTs) demonstrated markedly elevated levels of aspartate aminotransferase (AST, 3000 IU/L) and alanine aminotransferase (ALT, 1500 IU/L). Lactate dehydrogenase (LDH) was elevated to 1300 IU/L, consistent with ongoing haemolysis. Haptoglobin levels were decreased, and haemoglobinuria was noted. These findings raised concerns for an underlying haemolytic process. Additional tests of Epstein-Barr virus (EBV), cytomegalovirus (CMV), and hepatitis A virus (HAV) serology all tested negative, and procalcitonin was also negative. An ultrasound of the abdomen was initially done, and a degree of liver coarseness and gallbladder wall thickening was observed, associated with free fluid adjacent to the liver. Splenomegaly was also noted at 12 cm. A bone marrow aspiration was performed to rule out malignancy, and findings were consistent with peripheral platelet destruction without evidence of leukemia or lymphoma. Based on these findings, the patient was provisionally diagnosed with immune thrombocytopenic purpura (ITP) and treated with corticosteroids and intravenous immunoglobulin (IVIG). Despite therapy, symptoms, including abdominal distension and fatigue, persisted.

Over subsequent months, the patient’s condition progressively worsened, with worsening abdominal distension, jaundice, and bilateral lower limb edema. Repeat investigations two months later showed further deterioration (Table 2).

**Table 2:** Follow up laboratory findings two months after initial presentation.

Parameter	Value	Reference range	Interpretation
Aspartate aminotransferase (AST)	174 IU/L	10-40 IU/L	Elevated but subsided from the peak, indicating chronicity of liver injury
Alanine aminotransferase (ALT)	84 IU/L	7-56 IU/L	Elevated but subsided from the peak, indicating chronicity of liver injury
Albumin	2.2 g/dL	3.5-5.5 g/dL	Decreased, indicating liver dysfunction
Prothrombin time (PT)	49 sec	11-15 sec	Markedly prolonged
International normalized ratio (INR)	3.96	0.8-1.2	Markedly prolonged
Platelets	68×10³/µL	150-400×10³/µL	Worsening thrombocytopenia

Repeated abdominal ultrasound revealed a nodular liver contour, ascites, and splenomegaly increasing to 17 cm. A comprehensive diagnostic workup was undertaken to identify the underlying cause of liver dysfunction. The differential diagnosis included autoimmune liver disease, viral hepatitis, and metabolic disorders such as WD. Serological tests for autoimmune and viral hepatitis were negative. However, ceruloplasmin levels were markedly reduced (<0.08 mg/dL), and 24-hour urinary copper excretion was significantly elevated (400 µg/dL). Although Kayser-Fleischer rings were absent, these findings strongly suggested WD. The WD scoring system yielded a score of >4, confirming its diagnosis complicated by liver cirrhosis and haemolysis.

Management and clinical course

The patient was promptly initiated on copper-chelating therapy with D-penicillamine to enhance urinary copper excretion. Zinc sulfate was added to reduce gastrointestinal copper absorption. Additionally, vitamin supplementation (Vitamins E, K, and D) was provided to address deficiencies associated with liver dysfunction. Proton pump inhibitor therapy with pantoprazole was initiated to mitigate potential gastric complications.

Despite these interventions, the patient’s condition deteriorated. He was readmitted with hyponatremic dehydration and metabolic acidosis, raising concerns about potential renal involvement due to copper deposition. A nephrology consultation was obtained to evaluate for tubular dysfunction. The kidneys appeared normal in size with a slight increase in echogenicity on repeated ultrasound. Tubular dysfunction remained unconfirmed, and the kidney injury was associated with hypervolemic hyponatremia. Aldactone was started as the diuretic of choice. 

During hospitalization, the patient developed fever and abdominal pain, raising suspicion for spontaneous bacterial peritonitis (SBP). Empirical broad-spectrum antibiotic therapy with cefotaxime was initiated while awaiting culture results. The clinical course was further complicated by the onset of severe encephalopathy, with a Glasgow Coma Scale (GCS) score below 8, necessitating intubation and mechanical ventilation.

Subsequent complications included gastrointestinal bleeding and acute respiratory distress syndrome (ARDS). Laboratory evaluation revealed severe metabolic acidosis (bicarbonate 10 mEq/L), which was managed with intravenous sodium bicarbonate and fluid resuscitation. Coagulopathy was treated with fresh frozen plasma (FFP), and vasopressors (dopamine and dobutamine) were required to maintain haemodynamic stability. Persistent hypoglycemia necessitated continuous glucose infusion and fluid adjustments.

Despite aggressive multidisciplinary interventions, the patient's condition continued to decline. Massive pulmonary haemorrhage resulted in cardiopulmonary arrest, and resuscitation efforts were unsuccessful.

## Discussion

Wilson disease (WD) presents a significant diagnostic challenge, particularly in pediatric populations. The disease’s broad range of clinical manifestations often results in delayed diagnosis, as early signs may resemble those of other, more common conditions. This diagnostic complexity is exemplified in the present case, where the patient's initial symptoms of haemolytic anaemia and hepatosplenomegaly, along with elevated liver enzymes, were misattributed to immune thrombocytopenic purpura (ITP).

The initial misdiagnosis of ITP underscores the diagnostic difficulty of WD, especially in pediatric patients. ITP, a condition characterized by isolated thrombocytopenia, typically lacks specific organ involvement, including hepatosplenomegaly or haemolysis. The presence of these findings should have raised suspicion for an underlying systemic disorder. However, the absence of classic WD signs, such as neurological symptoms and Kayser-Fleischer rings, delayed the correct diagnosis. While neurological and ophthalmological manifestations are hallmark features of WD, they may not always co-occur with hepatic symptoms. Literature suggests that neurological signs often develop after hepatic manifestations, but hepatic involvement may exist independently or might precede neurological manifestations by decades [13].

Haemolysis in WD results from free copper release into the bloodstream, causing oxidative damage to red blood cells [14]. This pathophysiology bridges haemolysis and liver dysfunction, as significant hepatic injury leads to excessive copper release into the circulation. Elevated lactate dehydrogenase (LDH), low haptoglobin, and haemoglobinuria, as seen in this case, are hallmark indicators of haemolysis. Recognizing this relationship is critical, as it could have prompted earlier consideration of WD.

Hepatic manifestations of WD in pediatric patients can vary from jaundice, abdominal pain, and hepatomegaly to more subtle signs like isolated transaminitis or persistent elevations in liver enzymes [15]. These symptoms may mimic other conditions, such as autoimmune hepatitis or fatty liver disease, delaying diagnosis further. Conditions such as haemolytic uremic syndrome, hereditary spherocytosis, or even sepsis can mimic features of WD and should be considered during differential diagnosis. In rare cases, acute liver failure or cirrhosis can develop in very young children, with cases reported as early as 8 months of age [9]. This variability emphasizes the importance of considering WD in children with unexplained liver dysfunction.

Neurological symptoms, typically associated with WD, may emerge early in the disease and sometimes precede hepatic involvement. Prepubertal children can present with tremors, dystonia, dysarthria, or gait disturbances, which can be confused with other neurological conditions [16]. In some cases, psychiatric symptoms such as mood swings, behavioral issues, or depression may be the first signs, complicating the diagnosis by resembling primary psychiatric disorders. Additionally, rare clinical features, such as haemolytic anaemia, renal tubular acidosis, seizures, parkinsonism, and autonomic dysfunction, can further obscure the diagnosis. These atypical presentations highlight the diverse and sometimes misleading nature of WD.

With all the above, diagnosing WD in pediatric patients is essentially complicated by the variability and subtlety of clinical features. Kayser-Fleischer rings a hallmark of significant neurological involvement, may not be present in the early stages, as seen in this case. Biochemical markers like low ceruloplasmin levels and elevated 24-hour urinary copper excretion are essential for diagnosis, but their interpretation must be cautious due to potential confounding factors. Ceruloplasmin levels may be reduced in other conditions, including protein-losing enteropathies, nephrotic syndrome, or malnutrition, whereas elevated urinary copper can overlap with other hepatic disorders like autoimmune hepatitis or chronic cholestasis.

A comprehensive clinical history and thorough examination are critical for accurate diagnosis. Many children with WD may appear asymptomatic or present with mild, nonspecific symptoms despite underlying liver damage. This underscores the need for heightened vigilance and a systematic approach when evaluating pediatric patients with unexplained hepatic, neurological, or haematologic abnormalities. Recognizing the diverse presentations of WD is crucial for initiating timely and effective treatment, thus preventing severe complications.

This case demonstrates the severe consequences of delayed diagnosis. The patient’s progression to liver cirrhosis, spontaneous bacterial peritonitis (SBP), acute respiratory distress syndrome (ARDS), and multisystem organ failure reflects the untreated natural history of WD. Advanced liver disease predisposes patients to life-threatening complications, including portal hypertension, metabolic derangements, and infections such as SBP. Once significant liver damage occurs, the disease progresses rapidly, resulting in a poor prognosis.

The Wilson disease scoring system is a valuable tool for diagnosis, particularly in atypical cases. It combines clinical findings, biochemical markers, and genetic testing into a structured framework, assigning scores based on parameters such as serum ceruloplasmin, urinary copper excretion, presence of Kayser-Fleischer rings, and genetic mutations. A score ≥4 suggests a high likelihood of WD (Table 3). In this case, the score of >4 strongly indicated WD, underscoring the utility of this scoring system in guiding timely intervention. However, the system's limitations, such as the availability of genetic testing in resource-limited settings, must be acknowledged.

**Table 3:** Wilson disease scoring system. Total score: ≥4: diagnosis established, 3: diagnosis possible, more tests needed, ≤2: diagnosis very unlikely.

Test	Parameter	Score
Kayser-Fleischer ring	Present	2
	Absent	0
Neurological symptoms	Severe	2
	Mild	1
	Absent	0
Serum ceruloplasmin	Normal (>0.2 g/L)	0
	0.1-0.2 g/L	1
	<0.1 g/L	2
Coombs-negative haemolytic anaemia	Present	1
	Absent	0
Liver copper (in the absence of cholestasis)	>250 μg/g dry weight (>4 μmol/g)	2
	50–249 μg/g dry weight (0.8-4 μmol/g)	1
	Normal ≤50 μg/g dry weight (<0.8 μmol/g)	-1
	Rhodanine-positive granules	1
Urinary copper (in the absence of acute hepatitis)	Normal	0
	1-2 × ULN	1
	>2 × ULN	2
	Normal but >5×ULN after D-penicillamine	2
Mutation analysis of *ATP7B*	Biallelic deleterious variants	4
	One deleterious variant	1
	No mutation detected	0

The diagnostic workup in this case ultimately confirmed the diagnosis, with undetectable ceruloplasmin levels (<0.08 mg/dL) and markedly elevated 24-hour urinary copper excretion (400 µg/dL), both strongly indicative of WD. Preventive strategies and early detection could have significantly altered the outcome in this case. Routine screening for WD in patients with unexplained liver dysfunction, especially in high-risk populations or those with a family history of WD, is crucial. Raising awareness among healthcare providers about the diverse clinical presentations of WD can improve diagnostic accuracy. Additionally, prompt initiation of copper-chelating therapy or preemptive liver transplantation in advanced cases can prevent irreversible complications.

Management of WD depends on the stage of the disease. In early and moderate cases, copper-chelating agents such as D-penicillamine or trientine promote copper excretion, while zinc therapy is used to reduce copper absorption [17]. In advanced cases like the one described here, liver transplantation remains the definitive treatment to prevent further complications and improve survival. Unfortunately, despite the initiation of chelation therapy, the rapid progression of this patient’s disease precluded a favourable outcome.

This case highlights several critical lessons for clinicians. WD should always be considered in the differential diagnosis of pediatric patients with unexplained liver dysfunction, haemolysis, or hepatosplenomegaly, even in the absence of classic neurological symptoms or Kayser-Fleischer rings. Early recognition and intervention are essential to prevent irreversible liver damage and life-threatening complications. Comparing this case to existing literature highlights the potential for ITP, autoimmune hepatitis, or haemolytic syndromes to mimic WD, further emphasizing the importance of a systematic diagnostic approach. Enhanced awareness of such mimickers can minimize delays in diagnosing WD.

Despite the tragic outcome in this case, it underscores the importance of early recognition and treatment in WD. Educational initiatives, increased utilization of the Wilson disease scoring system, and improved access to diagnostic tools are critical steps toward reducing morbidity and mortality associated with this challenging disorder.

## Conclusions

This case highlights the diagnostic challenges of WD in pediatric patients, particularly when it presents with atypical features like haemolytic anaemia and hepatosplenomegaly without the classical signs of liver failure or neurological involvement. The delayed diagnosis in this case led to severe liver damage and organ failure, underscoring the need for early biochemical testing and a broader awareness of WD’s diverse manifestations.

The use of the WD scoring system can aid in diagnosis, even when classic features are absent. This case emphasizes the importance of a multidisciplinary approach involving hepatologists, neurologists, and intensivists in managing advanced WD. Furthermore, it highlights the need for better early detection methods, such as potential biomarkers or advanced imaging techniques. Continuous education on rare diseases like WD is essential to avoid misdiagnosis and improve patient outcomes.

## References

[1] (2025). Wilson disease. https://medlineplus.gov/genetics/condition/wilson-disease/.

[2] (2025). Hepatolenticular degeneration. https://www.cancer.gov/publications/dictionaries/cancer-terms/def/hepatolenticular-degeneration.

[3] (2025). Wilson disease: practice essentials, background, etiology. https://emedicine.medscape.com/article/183456-overview.

[4] Hermann W (2019). Classification and differential diagnosis of Wilson's disease. Ann Transl Med.

[5] (2025). In the beginning: neonatal screening and management of Wilson disease. https://www.aasld.org/liver-fellow-network/core-series/clinical-pearls/beginning-neonatal-screening-and-management-wilson.

[6] Schilsky ML (2014). Wilson disease: Clinical manifestations, diagnosis, and treatment. Clin Liver Dis (Hoboken).

[7] Stremmel W, Merle U, Weiskirchen R (2019). Clinical features of Wilson disease. Ann Transl Med.

[8] Immergluck J, Anilkumar AC (2024). Wilson Disease. https://www.ncbi.nlm.nih.gov/books/NBK441990/.

[9] Poujois A, Woimant F (2019). Challenges in the diagnosis of Wilson disease. Ann Transl Med.

[10] Martínez-Morillo E, Bauça JM (2022). Biochemical diagnosis of Wilson's disease: an update. Adv Lab Med.

[11] Volpert HM, Pfeiffenberger J, Gröner JB (2017). Comparative assessment of clinical rating scales in Wilson's disease. BMC Neurol.

[12] Shribman S, Warner TT, Dooley JS (2019). Clinical presentations of Wilson disease. Ann Transl Med.

[13] Jopowicz A, Tarnacka B (2023). Neurological Wilson's disease signs-hepatic encephalopathy or copper toxicosis?. Diagnostics (Basel).

[14] Kasztelan-Szczerbinska B, Cichoz-Lach H (2021). Wilson's disease: an update on the diagnostic workup and management. J Clin Med.

[15] Ghosh U, Sen Sarma M, Samanta A (2023). Challenges and dilemmas in pediatric hepatic Wilson's disease. World J Hepatol.

[16] Page S, Shaik L, Singh R, Rathore SS, Shah K (2020). Neuropsychiatric atypical manifestation in Wilson's disease: a case report and literature review. Cureus.

[17] Hedera P (2019). Clinical management of Wilson disease. Ann Transl Med.

